# A Case of Sandwich Repair for Posterior Ventricular Septal Rupture through the Right Atrium

**DOI:** 10.70352/scrj.cr.25-0457

**Published:** 2025-10-18

**Authors:** Shunsuke Nakata, Hideki Ito, Rena Usui, Shiori Kako, Akihiko Usui

**Affiliations:** 1 Cardiovascular Surgery, Fujita Health University Okazaki Medical Center, Okazaki, Aichi, Japan; 2 Department of Cardiac Surgery, Nagoya University Graduate School of Medicine, Nagoya, Aichi, Japan

**Keywords:** ventricular septal rupture, myocardial infarction, surgical approach

## Abstract

**INTRODUCTION:**

We encountered a case in whom ventricular septal rupture (VSR) repair was performed only through right atriotomy. A few cases of VSR repair using the right atrial approach have been reported in the literature. We report our experiences with focusing on the surgical technique.

**CASE PRESENTATION:**

The patient was a 79-year-old woman who was admitted to the emergency room with dizziness and vomiting. Echocardiography showed inferior myocardial infarction and posterior VSR. Emergency percutaneous coronary artery intervention was performed on the proximal right coronary artery and recanalization was achieved. Delayed surgery was planned because of the stability of her hemodynamics. Sandwich repair using two bovine pericardial patches was performed only through right atriotomy on the 14th day of hospitalization under intra-aortic balloon pumping (IABP). The location of the VSR was identified by saline injection through the left ventricular vent. A VSR 18 mm in length was observed after resection of several trabeculae. A 3.5 × 2.5 cm oval patch of bovine pericardium was placed on the left side of VSR and another patch was attached to the right side to cover the VSR with 8 pieces of monofilament-interrupted U sutures. She was discharged from the hospital on the 108th POD after long ventilatory management and temporary hemodialysis.

**CONCLUSIONS:**

VSR of the posterior interventricular septum due to inferior myocardial infarction is a good candidate for the right atrial approach. It is important to diagnose the location and shape of the VSR in advance. The location of the VSR can be identified by saline injection through the left ventricular vent and resection of several trabeculae is essential to expose the whole VSR. The right atrial approach can minimize impairment of the ventricular function and may be a promising approach for VSR repair.

## Abbreviations

IABP
: intra-aortic balloon pumping
VSR
: ventricular septal rupture

## INTRODUCTION

Post-infarction VSR is a serious complication of myocardial infarction, and its surgical outcomes need to be improved. In the 2020 annual report of the Japanese Association for Thoracic Surgery, the 30-day mortality rate of VSR repair was high, at 26.8%, with no clear improvement from 30.4% in 2010.^1,2)^ VSR repair generally results in significant surgical damage, in addition to impairment of the cardiac function caused by acute myocardial infarction. VSR repair generally requires ventriculotomy, which further worsens cardiac dysfunction and makes it difficult to improve surgical outcomes. We encountered a case in whom VSR repair was performed only through right atriotomy. A few cases of VSR repair using the right atrial approach have been reported.^3–6)^ We report our experiences and focus on the surgical technique.

## CASE PRESENTATION

**Fig. 1:**
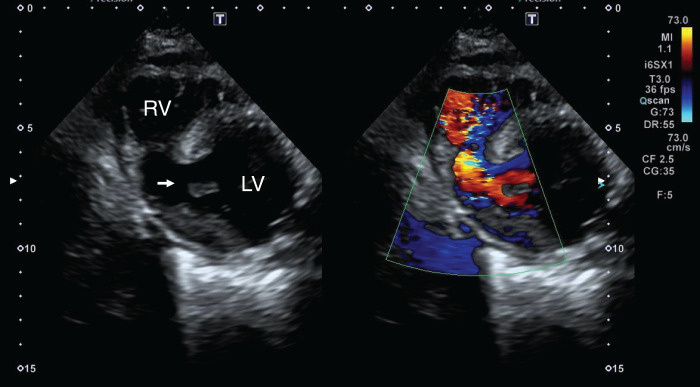
Echocardiography trans-thoracic echocardiography revealed decreased right ventricular contractility and a left-to-right shunt through a posterior interventricular septal rupture (arrow). LV, left ventricle; RV, right ventricle

**Fig. 2:**
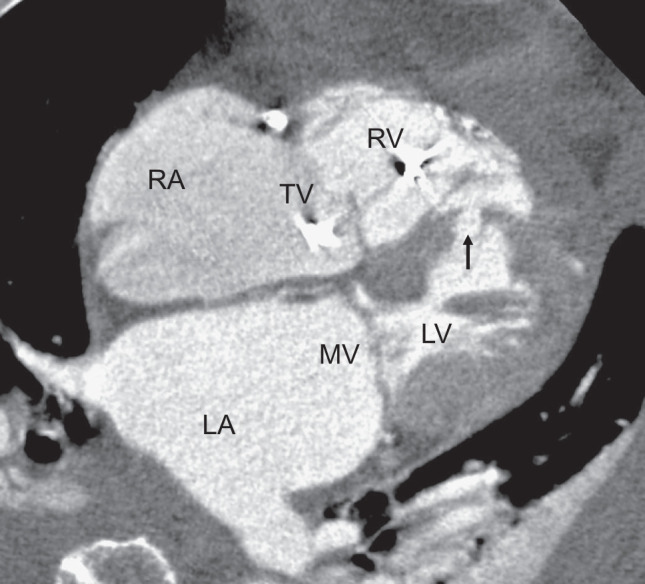
Cardiac CT. A 4-chamber cardiac CT image showed a myocardial defect at the posterior wall of the interventricular septum (arrow). The VSR was located near the apex and was kept away from the subvalvular apparatus of the mitral and tricuspid valves. LA, left atrium; LV, left ventricle; MV, mitral valve; RA, right atrium; RV, right ventricle; TV, tricuspid valve; VSR, ventricular septum rupture

A 79-year-old woman was admitted to the emergency room with complaints of dizziness and vomiting. Electrocardiography revealed ST elevation in II, III, and aVf. Echocardiography revealed decreased right ventricular contractility and a left-to-right shunt through the posterior VSR (**Fig. 1**). Emergency coronary arteriography revealed total occlusion of the proximal right coronary artery, while no significant stenosis was observed in any other coronary arteries, and percutaneous coronary artery intervention was performed to achieve recanalization using drug-eluting stents (Resolute Onvx; Medtronic, Minneapolis, MN, USA). Although echocardiography revealed left to right shunt with 2.26 of pulmonary blood flow/systemic blood flow ratio (Qp/Qs), the hemodynamics were relatively stable, and delayed surgery was planned under IABP, medical management, and anti-platelet therapy with aspirin and prasugrel. Her blood creatine kinase level peaked at 3685 U/L on the 2nd day of hospitalization. Cardiac CT showed a myocardial defect (length, 15 mm; width, 10 mm) located near the apex of the posterior interventricular septum covered with trabeculae, and the VSR was kept away from the subvalvular apparatus of the mitral valve and tricuspid valve (**Fig. 2**). Right heart catheterization on the 10th day of hospitalization revealed pulmonary artery pressure (PA) 42/18/30, pulmonary capillary wedge pressure (PCWP) 16, right atrium pressure (RA) 13, and Qp/Qs 3.24.

### Surgical technique

**Fig. 3:**
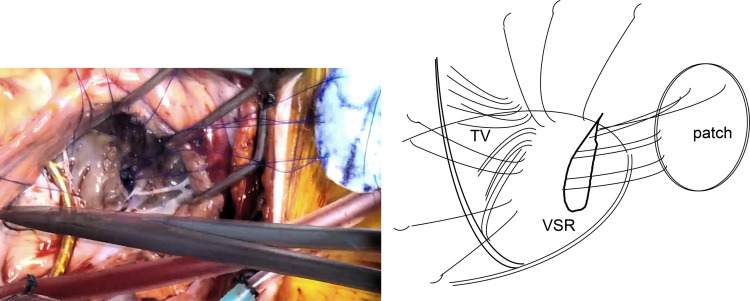
Operative findings 1. VSR of 18 mm in length was observed. A 3.5 × 2.5 cm oval patch of bovine pericardium attached to Teflon felt was made. Eight 3-0 SH polypropylene interrupted U sutures were first inserted into the bovine pericardial patch and then placed around the VSR with full-thickness, wide bites from the left ventricular side to the right ventricular side. TV, tricuspid valve; VSR, ventricular septum rupture

**Fig. 4:**
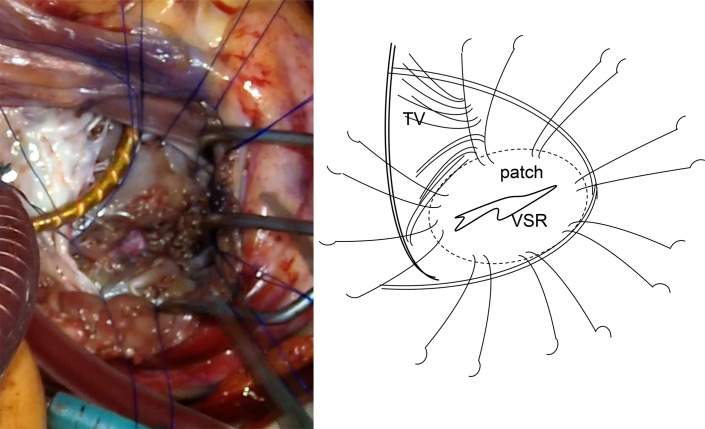
Operative findings 2. A bovine patch was inserted through the VSR and placed on the left side of the intraventricular septum. TV, tricuspid valve; VSR, ventricular septum rupture

**Fig. 5:**
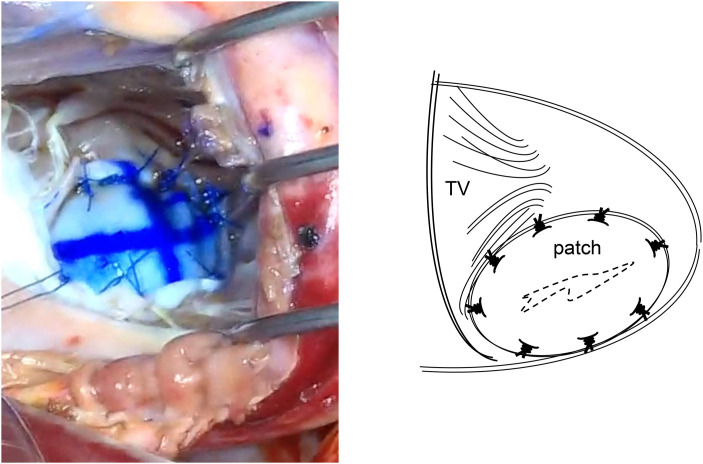
Operative findings 3. Another 3.5 × 2.5 cm oval bovine pericardial patch was attached to the right ventricular side and fixed with the same U sutures to cover the VSR. TV, tricuspid valve; VSR, ventricular septal rupture

Prasugrel was terminated at 6 days, aspirin was stopped, and heparin was administered continuously for 3 days before the operation under heart team discussion. VSR repair was performed on the 14th day of hospitalization. We considered that the tissue fragility may have improved at this time. Therefore, a sandwich repair using two bovine pericardial patches was performed only through right atriotomy (**Supplementary Video**). The heart was exposed via median sternotomy. Cardiopulmonary bypass was established via cannulation of the ascending aorta and bicaval cannulation, and a left ventricular vent was placed through the right superior pulmonary vein. Pulmonary vein isolation was performed with cryoIce cryoablation (AtriCure; Mason, WV, USA), and the left appendage was resected with endo GIA (Medtronic) for atrial fibrillation before aortic cross-clamping. Myocardial protection was achieved in antegrade cold blood cardioplegia under aortic cross-clamping. The right atrium was obliquely opened and the tricuspid valve leaflets were retracted to expose the posterior ventricular septum. The location of the VSR was hard to find and identified by saline injection through the left ventricular vent. Several trabeculae were resected to expose the entire VSR and a myocardial defect of 18 mm in length was observed (**Fig. 3**). The area of the myocardial infarction was relatively firm. A 3.5 × 2.5 cm oval patch of bovine pericardium attached to Teflon felt was made. Eight 3-0 SH polypropylene interrupted U sutures were first inserted into the left ventricular pericardial patch and then placed around the VSR with full-thickness, wide bites from the left ventricular side to the right ventricular side (**Fig. 4**). After insertion of the left ventricular patch through the VSR, another 3.5 × 2.5 cm oval bovine pericardial patch was attached to the right ventricular side and fixed with the same U sutures to cover the VSR (**Fig. 5**). The absence of patch leak was also confirmed with saline injection via the left ventricular vent. Ring annuloplasty of the tricuspid valve was performed, and a water test revealed little tricuspid valve regurgitation. The operation was completed by closing of the right atrial wall. The pump was easily weaned under an IABP. The aortic cross-clamp time, cardiopulmonary bypass time, and operation time were 137, 213, and 402 min, respectively.

### Postoperative course

Postoperatively, she required long ventilatory management and tracheotomy was performed on the 14th POD and ventilation was weaned on the 63rd POD. She also required hemodialysis temporarily due to acute renal failure. However, she was discharged from the hospital 108 days after operation. Postoperative echocardiography revealed no VSR leakage or tricuspid valve regurgitation.

## DISCUSSION

VSR repair is generally performed through the left ventricular approach, and right ventricular approaches have recently been reported.^7)^ Right ventricular dysfunction was reported to occur in 40% of cases of myocardial infarction and in nearly all cases of inferior myocardial infarction.^8)^ Therefore, it is ideal to avoid ventriculotomy and perform VSR repair only through right atriotomy, especially for inferior myocardial infarction. However, several technical improvements are required to perform VSR repair only through the right atrial approach.

In the right ventricle, trabeculation was extensively developed, making it challenging to fully visualize the entire anatomy of the VSR. Additionally, it was difficult to clearly delineate the boundary between the myocardial infarction and the healthy myocardium on the right side of the ventricular septum. Furthermore, the tricuspid valve or mitral valve can complicate VSR repair, and there is a risk of developing regurgitation of tricuspid valve or mitral valve following the procedure.

The injection of saline from the left ventricular vent is useful for identifying the location of the VSR, and some trabeculae should be resected to expose the whole VSR. Because the margins of the myocardial infarction are ill-defined on the right side of the intraventricular septum, a suture line should be established at a sufficient distance from the edge of the VSR. It is also important to diagnose the area of myocardial infarction in advance. Multiplane reconstruction of cardiac CT is useful for determining the morphology of the VSR and area of myocardial infarction. It is also important to fully evaluate the relationship between the VSR and the mitral valve, tricuspid valve or their subvalvular apparatus. Cardiac CT findings can provide surgeons with precise information regarding the positional relationship between them. Cardiac CT can be performed in a short time without invasion and provides important information, including information about coronary artery disease. Even in emergencies, it should be performed when possible.

To ensure complete resection of necrotic tissue around the VSR, meticulous attention must be paid not to injure the mitral valve, tricuspid valve, or their subvalvular apparatus. In the present case, the VSR was away from the tricuspid septal leaflet, and VSR repair can be performed without causing tricuspid valve regurgitation. However, in cases where the VSR is close to the tricuspid septal leaflet, cutting and repairing the chorda of the septal leaflet may be necessary. We experienced a case with repair involving creation of a neochord from a bovine pericardial patch concomitant with tricuspid annuloplasty.

The timing of surgery is another concern of VSR repair. Myocardial infarction sites become progressively fibrotic over time, reducing tissue fragility. Therefore, some attempts have been made to delay the timing of surgery using IABP, Impella (Abiomed, Danvers, MA USA) or mechanical cardiac support. The 2023 European acute coronary syndrome guidelines recommend a more nuanced approach: prompt surgery for patients with refractory shock or persistent right ventricle dysfunction, and a delayed approach in the remaining patients, if possible beyond day 7 after diagnosis.^9)^ In the present case, delayed surgery was planned, and surgery was performed on the 14th day after admission. The fragility of the myocardial infarction area was relatively improved at this time. In the right atrial approach, all sutures are required through the VSR. There is a risk of expansion of the VSR when tissue fragility is severe. Therefore, early surgery may not be suitable because of the high tissue fragility, and delayed surgery may be preferable for the right atrial approach.

The right atrial approach is well suited to posterior septal VSR associated with inferior wall infarction but it is difficult to apply to anterior septal VSR. We believe that the following are good conditions for the right atrial approach: limited infarct area, relatively recovered tissue vulnerability, and VSR located on the apical side, away from the mitral and tricuspid valves. The mitral valve cannot be observed through the VSR, while the tricuspid valve can be observed under direct vision; therefore, a VSR that is close to the mitral valve and its subvalvular apparatus is considered as a contraindication for the right atrial approach.

## CONCLUSIONS

VSR of the posterior interventricular septum due to inferior myocardial infarction is a good candidate for the right atrial approach. It is important to diagnose the location and shape of the VSR in advance by using cardiac CT. The location of the VSR can be identified by saline injection through the left ventricular vent, and resection of several trabeculae is essential for exposing the entire VSR. The right atrial approach can minimize the impairment of ventricular function and bleeding. Thus, this may be a promising approach for VSR repair.

## SUPPLEMENTARY MATERIALS

Supplementary Video Supplementary video shows surgical procedure of sandwich repair of VSR only through right atriotomy.

## DECLARATIONS

### Availability of data and materials

The data are available from the corresponding author upon reasonable request.
